# Implementation of robotic gynecological surgery in a German University Hospital: patient safety after 110 procedures

**DOI:** 10.1007/s00404-020-05751-8

**Published:** 2020-08-25

**Authors:** Dimitrios Balafoutas, Achim Wöckel, Christine Wulff, Ralf Joukhadar

**Affiliations:** Department of Obstetrics and Gynecology, University Hospital of Würzburg, Josef-Schneider-Str. 4, 97080 Würzburg, Germany

**Keywords:** Robotic hysterectomy, Robotic sacrocolpopexy, Implementation, Robotic complications, Polyvinylidene fluoride (PVDF)

## Abstract

**Purpose:**

Robotic surgery represents the latest development in the field of minimally invasive surgery and offers many technical advantages. Despite the higher costs, this novel approach has been applied increasingly in gynecological surgery. Regarding the implementation of a new operative method; however, the most important factor to be aware of is patient safety. In this study, we describe our experience in implementing robotic surgery in a German University Hospital focusing on patient safety after 110 procedures.

**Methods:**

We performed a retrospective analysis of 110 consecutive robotic procedures performed in the University Hospital of Würzburg between June 2017 and September 2019. During this time, 37 patients were treated for benign general gynecological conditions, 27 patients for gynecological malignancies, and 46 patients for urogynecological conditions. We evaluated patient safety through standardized assessment of intra- and postoperative complications, which were categorized according to the Clavien–Dindo classification.

**Results:**

No complications were recorded in 90 (81.8%) operations. We observed Clavien–Dindo grade I complications in 8 (7.3%) cases, grade II complications in 5 (4.5%) cases, grade IIIa complications in 1 case (0.9%), and grade IIIb complications in 6 (5.5%) cases. No conversion to laparotomy or blood transfusion was needed.

**Conclusion:**

Robotic surgery could be implemented for complex gynecological operations without relevant problems and was accompanied by low complication rates.

## Introduction

Since the federal drug administration (FDA) approval of the daVinci system for gynecological operations in 2005, an increasing number of minimally invasive procedures have been performed robotically [1]. The main benefits of the system are the technical advantages like the visualization with high definition, three-dimensional stereo-sight, and the open surgical orientation during the instrument movement. Additionally, the design of the EndoWrist instruments enables intuitive precision movements along with tremor filtering. These technical advantages seem to improve surgical outcome, especially in cases of challenging anatomy or obesity [2]. Moreover, the robotic system seems to make endoscopic approach possible in highly complex cases, thus, reducing the need for laparotomy [3, 4]. Data suggest its advantages for enucleation of deep uterine fibroids [5], reconstruction of multicompartmental pelvic floor defects [6], and minimally invasive surgery of endometrial cancer, especially in obese patients. In such cases, robotic surgery can reduce intraoperative blood loss and leads to shorter postoperative hospitalization and sick leave in comparison to conventional laparoscopy [7].

In Germany, an increase in the availability of the robotic systems could be observed in the recent years [8]; however, the utilization in gynecological surgery remains relatively scarce. The main reasons for this seem to be the increased costs of obtaining and running the system, as well as the logistic obstacles of the implementation of the novel technique. These challenges are mainly healthcare system dependent in terms of reimbursement structure and numeral calculation of needed hospital staff. Therefore, we believe that the reports from American centers with yearlong experience in robotic surgery do not directly apply to the German health care system.

Whereas one important factor from practical perspective is the learning curve; patient safety remains the most important issue to look for from an ethical as well as a forensic perspective. Although robotic surgery is widely accepted because of the technological advantages, there is lack of reports about the implementation of this technique in gynecological departments in German hospitals. Our goal was to address this issue and to share our experience with colleagues aiming to perform the transition to robotic surgery.

## Patients/materials and methods

The four-arm robotic surgical system daVinci Xi in the two-console configuration with a simulation module was installed in 2017 at the University Hospital of Würzburg (Intuitive Surgical, Sunnyvale, California, United States).

### Patient selection

From July 2017 to September 2019, we performed 110 robotic gynecological operations. The indications were according to German and international guidelines [9–11] and comprised of general gynecological surgery (37 patients), malignant tumors (27 patients), as well as urogynecological conditions (46 patients). The mean patient age was 55 years, while prolapse patients tended to be older (Table 1). During the patient recruitment, experience with the system was taken into consideration. At the initial phase of the implementation, we mostly performed operations with lower expected complication rates and, subsequently, more challenging procedures. Additionally, within each group of surgical indications, we initially selected less complex cases and subsequently cases with prior abdominal surgery, patient BMI over 30, or expected difficult intraoperative anatomy. After performing the first 40 procedures, we mainly focused on selecting complex cases, which would pose difficulties in classical laparoscopy.

**Table 1:** Patient characteristics: age and body mass index (BMI) as mean ± standard deviation; ASA as percent in the corresponding groups

Parameter	All	Hysterectomy	Prolapse surgery	Rest
Number of patients (*n*)	110	52	44	14
Age	55.0 ± 12.3	51.9 ± 11.9	61.3 ± 9.4	45.8 ± 11.7
BMI	26.5 ± 5.4	26.7 ± 5.6	26.6 ± 5.6	25.5 ± 5.6
ASA 1	19 (17.3%)	9 (17.3%)	5 (11.4%)	5 (35.7%)
ASA 2	80 (72.3%)	40 (76.9%)	31(70.5%)	9 (64.3%)
ASA 3	11 (10%)	3 (57.7%)	8 (18.2%)	0

### Perioperative setting

The preparation for surgery was performed according to hospital standards and always included a clinical examination, transvaginal ultrasound, and common laboratory testing. Hemoglobin was assessed 1 day before the procedure and on the first day after surgery. Other preoperative examinations, for instance magnetic resonance tomography, were performed as needed. In case of patients with urogynecological conditions, we additionally performed a preoperative urodynamic testing as well as pelvic floor and renal ultrasound.

Regarding intraoperative setting, the patient was placed in lithotomy Trendelenburg position with both arms attached to the torso. The position of the robot was at the right side of the patient. In cases, where three arms were needed (*n* = 67, 61%), the robotic arms 1–3 (distal) were utilized. In procedures with four robotic arms (*n* = 43, 39%), the camera was mounted on arm 3 (second from proximal). Depending on the procedure, we used either the Hohl uterine manipulator (total hysterectomy) [12] or the Valtchev manipulator (urogynecological procedures) [13]. We placed the camera trocar at the umbilicus in most cases. In cases of three-arm procedures, the additional daVinci trocars were placed at the level of the umbilicus to the right and left from midline at a distance of about 6–9 cm. During four-arm procedures, the fourth trocar was placed at the left side laterally. In 105 (95.5%) procedures, an additional conventional trocar was placed at the left subcostal region.

The most widely used daVinci instruments were the Hot Shears™ (monopolar curved scissors), the Fenestrated Bipolar Forceps, and the Mega SutureCut™ Needle Driver. When transvaginal tissue extraction was not possible, the Alexis Contained Extraction System (Applied Medical, Rancho Santa Margarita, California, United States) was used. We performed the closure of the vaginal cuff in all procedures involving a total hysterectomy with Vicryl interrupted sutures (Polyglactin 910 coated Vicryl suture; Ethicon/Johnson & Johnson, New Brunswick, United States). The mesh material used in robotic sacrocolpopexy was the DynaMesh tailored implant (PR visible or PRS visible) made up of Polyvinylidenfluorid (PVDF), (FEG Textiltechnik mbH, Aachen, Germany), which was fixed with Ethibond sutures (Ethicon/Johnson & Johnson, Somerville, New Jersey, United States). Sentinel lymph nodes in patients with endometrial cancer were detected using the indocyanine green fluorescence (ICG). To do so, we injected the fluorescent dye (Verdye 5 mg/ml, Diagnostic Green, Aschheim, Germany) submucosally in the uterine cervix with a 23G × 1 ½″ needle and the lymph nodes were detected with the near-infrared camera of the daVinci Xi surgical system (firefly mode). The application was according to the FIRES Study by injecting 2 × 0.5–1 mL at 9 and 3 h of the cervix at a dose of 2.5 mg/mL [14–16].

### Postoperative follow-up

We controlled the patients postoperatively according to the hospital standards for the equivalent laparoscopic procedures. All urogynecological and oncological patients were examined in our outpatient department. The rest of the patients were contacted by telephone to register possible postoperative complications and reevaluation was offered in case of any symptoms. All patients could successfully be contacted postoperatively. The complications were categorized according to the standardized Clavien–Dindo classification [17], which is a well-validated tool for gynecological surgery [18] (Table 2).

**Table 2:** The Clavien–Dindo classification of surgical complications

Grade	Definition
Grade I	Any deviation from the normal postoperative course without need for intervention (exclusions: antiemetics, antipyretics, analgesics, diuretics, electrolytes, physiotherapy, and wound infections treated at the bedside)
Grade II	Pharmacological treatment other than grade I
Grade III	Need for surgical, endoscopic, or radiological intervention
Grade IIIa	No general anesthesia required
Grade IIIb	General anesthesia required
Grade IV	Life-threatening complications requiring ICU
Grade IVa	Single-organ dysfunction
Grade IVb	Multi-organ dysfunction
Grade V	Death

Dindo et al. Annals of Surgery 2004 [17]

### Collection of data and evaluation

We collected the data retrospectively using the hospital electronic documentation system. Additionally, we obtained the American Society of Anesthesiologists (ASA) physical status classification system from the anesthesia protocol. The day of surgery was calculated as day 1 of hospitalization. The follow-up data were obtained from the digital patients’ records. For those who were contacted only via telephone, an additional electronic entry was added in the records. After anonymization, the data were imported into Microsoft Excel 2016 (Microsoft Corporation, Redmond, Washington, United States) spreadsheets. For the descriptive statistics, we calculated for normally distributed variables the mean and standard deviation, whereas for variables non-normally distributed, we calculated the median and the interquartile range.

## Results

Regarding the indications for surgery, the most common one was the genital prolapse (*n* = 47, 42.7%) followed by uterine fibroids (*n* = 22, 20%) and endometrial cancer (*n* = 12, 10.9%) (Table 3). Upon consideration of the distinctive surgical steps, the most common procedure was the total hysterectomy (52 cases), either as a stand-alone operation or in the context of another operation. Other frequent procedures were salpingo-oophorectomy, supracervical hysterectomy, and prolapse surgery.

**Table 3:** Surgical indications and distinctive surgical steps

Indication for surgery	
Genital prolapse	47 (42.7%)
Uterine fibroids	22 (20%)
Endometrial cancer	12 (10.9%)
Deep infiltrating endometriosis	10 (9%)
High-grade cervical dysplasia	8 (7.3%)
Ovarian neoplasms (i.e., borderline)	4 (3.6%)
Endometrial intraepithelial neoplasia	3 (2.7%)
Metrorrhagia	2 (1.8%)
Cervical cancer	2 (1.8%)
Distinctive surgical steps	
Total hysterectomy	52
Salpingo-oophorectomy	22
LASH (laparoscopic-assisted supracervical hysterectomy)	17
Hysterosacropexy	16
Cervicosacropexy	15
Colposacropexy	11
Adhesiolysis	9
Staging	3
Pelvic sentinel lymph-node biopsy (ICG)	3
Pectopexy	2

The mean skin incision to closure time was 149 min. The hospitalization ranged from 2 to 15 days with a median stay of 4 days for the entire collective. We did not perform any outpatient robotic procedures. The mean blood loss (subjective estimate of the surgeon) was 38 ± 33 mL and the mean decrease in hemoglobin 1.5 ± 0.9 mg/dL (Table 4).

**Table 4:** Intra-and perioperative parameters: duration of surgery (minutes), estimated blood loss (mL), decrease in hemoglobin (mg/dL), and hospital stay (days)

Parameter	All	Hysterectomy	Prolapse surgery	Other
Duration	149 ± 33	130 ± 30	180 ± 39	119 ± 29
Blood loss	38 ± 33	40 ± 34,6	50 ± 33	25 ± 30
Hemoglobin decrease	1.5 ± 0.9	1.5 ± 1.1	1.4 ± 0,8	1.6 ± 1.0
Hospital stay	4 (4–5)*	4 (4–5)*	4 (4–5)*	4 (4–5)*

Results as shown as mean ± standard deviation

*Median (Q1–Q3)

No intraoperative complications occurred, as all complications were diagnosed postoperatively, either during hospital stay or after patient discharge. While 90 (81.8%) of the procedures were completed without a complication, there was need for a surgical revision under general anesthesia in six (5.5%) patients. The remaining 12.7% of the patients experienced grade I–IIIa complications (Table 5).

**Table 5:** Complications according to the Clavien–Dindo classification during the initial 110 robotic operations

Complication	*n*	Intervention
No complication	90 (81.8%)	No surgical intervention
Grade I	8 (7.3%)	
Grade II	5 (4.5%)	
Grade IIIa	1 (0.9%)	Surgical revision or need for intensive care
Grade IIIb	6 (5.5%)	
Grade IVa	0	
Grade IVb	0	
Grade V	0	

The recorded complications were classified according to the Clavien–Dindo method as follows:

### Complications classified as Clavien–Dindo grade I

We recorded grade I complications in eight cases: five hysterectomies, two sacropexies, and one case with deep infiltrating endometriosis. Such complications included hyperemesis, postoperative fever, increased need for pain medication, and temporary subileus symptoms. In one case with a gastrointestinal infection with norovirus, the hospital stay was prolonged to 9 days.

### Complications classified as Clavien–Dindo grade II

This type of complication was observed in five cases: three hysterectomies and two sacropexies. One patient had a hypertensive crisis and, in four cases, a postoperative infection had to be treated with intravenous antibiotics without the necessity of an operative intervention.

### Complications classified as Clavien–Dindo grade IIIa

A 53-year-old patient who underwent total hysterectomy and salpingo-oophorectomy because of cervical intraepithelial neoplasia grade 3 was admitted to the hospital on the 7th day after surgery with fever. We diagnosed a small wound site abscess at the vaginal cuff. The patient was treated with intravenous antibiotics and local draining of the abscess transvaginally under local anesthesia. The hospital stay was prolonged to 15 days in this particular case.

### Complications classified as Clavien–Dindo grade IIIb

Overall six cases with grade IIIb complications were observed: two cases with vaginal cuff dehiscence after a hysterectomy (1.8%), one case of a small vaginal mesh erosion (0.9%), two cases with postoperative hematoma (1.8%), and one case with a pelvic lymphocele (0.9%). From the complications mentioned above, four were diagnosed after discharge from the hospital, while in two cases, the diagnosis was made during postoperative hospital stay.

Details of the mentioned complications were as follows:

- A 41-year-old patient who had a total hysterectomy to treat a high-grade cervical intraepithelial neoplasia was admitted 6 weeks postoperatively with a vaginal cuff insufficiency after sexual intercourse. The complication was managed by laparoscopic revision of vaginal stump.
- A 63-year-old patient presented after robotic hysterectomy for endometrial cancer with a persistent vaginal discharge due to partial vaginal cuff insufficiency. She received a laparoscopic closure of the vaginal cuff to treat the situation.
- A 61-year-old patient was admitted with increased vaginal discharge 4 weeks after a robotic sacropexy due to a small-sized (4 × 2 mm) mesh erosion in the posterior fornix, which was eventually corrected vaginally without the need for general anesthesia.
- A 62-year-old patient developed a postoperative hematoma at the right abdominal wall on the first postoperative day after robotic colposacropexy along with extensive adhesiolysis of the bowel. At the laparoscopic revision, no definitive source of bleeding could be identified.
- A 41-year-old patient developed a hemoperitoneum after a robotic hysterectomy performed to treat large uterine fibroids. The revision was performed laparoscopically. In the succeeding laboratory tests, an undetected factor-VIII deficiency was diagnosed.
- A 73-year-old patient developed a lymphocele 8 weeks after a robotic pectopexy, during which an enlarged pelvic lymph node (incidental finding) was removed. The lymphocele was fenestrated laparoscopically.

**Fig. 1:**
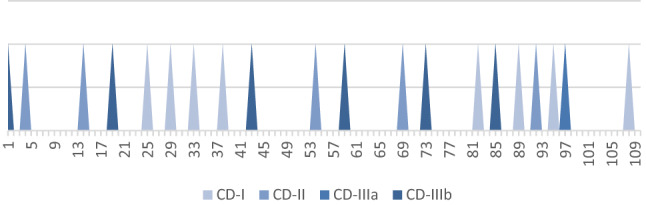
Distribution of the perioperative complications according to the Clavien–Dindo classification with increasing number of operations. CD Clavien–Dindo grade

Furthermore, we examined the frequency of complications in regard to surgical experience and found it to be evenly distributed over time without a tendency of cumulated occurrence during the initial implementation phase (Fig. 1).

Fortunately, we did not observe trocar-specific complications, injuries to bladder or bowel, need for intra- or postoperative blood transfusion, or the necessity for conversion to laparotomy.

## Discussion

This study represents one of the few reports on robotic surgery in gynecology in German hospitals and, to our knowledge, the first description of the implementation process of this new technique for gynecological surgery focusing on patient safety. In this report, we describe our proceedings and report patient safety during the implementation and the initial 110 robotic procedures.

An ongoing trend towards minimally invasive surgery can be observed in the last years. Experience from the United States demonstrates that the robotic systems further revolutionized gynecologic surgery raising minimally invasive feasibility and improving surgical outcome in both gynecological oncology and complex benign conditions [2, 3]. The technological advantages of the daVinci system, in particular the free orientation and movement of instruments along with the excellent three-dimensional visualization, provide an unprecedented precision during minimally invasive surgery. Due to the intuitive control instruments, the learning curve seems to be steeper than the one of the conventional laparoscopy. However, mainly because of the higher costs, this technology is commonly used for the more complex gynecological operations [19].

Yet, it has to be kept in mind that the selection of these more complex procedures to be performed using this surgical system bears some risks as complex operations are prone to complications, regardless which route of surgical approach is chosen [20]. Therefore, it is of utmost importance that patient safety is ensured by strict monitoring of all complications, especially during the implementation of the new technique. Although there are a few prospective randomized studies comparing the perioperative complications of robotic surgery with those in laparoscopic procedures, many large retrospective data are available that confirm the equivalence of robotic surgery with laparoscopy concerning complication rates [21, 22].

One main obstacle in comparing data from many studies is the methodic diversity in registering the complications and evaluating them. Therefore, the Clavien–Dindo classification, which offers a standardized tool for recording and evaluating surgical complications, was selected in this study. Concerning the follow-up period of 12 weeks, we believe that it is adequate to evaluate the impact of the operative method on potential complications. It has been shown that in cases of a vaginal cuff insufficiency, the diagnosis is mostly made within 2 months after the operation [23]. On the other hand, implant erosions after a sacropexy would need a much longer follow-up period, because most erosions develop in the long term.

Among all major postoperative complications in this study that had required surgical revision, both the vaginal stump insufficiency and the postoperative hematomas occurred more than once, whereas the other major complications were sporadic and showed no tendency for repetition. Yet, it has to be mentioned that one of the patients had a factor-VIII insufficiency, which independently increases the risk of postoperative hematomas.

A closer look at the other major complications reveals that the early postoperative erosion of the implant and the lymphocele seem to be independent from the operative mode. Erosions after a sacropexy represent one of the most frequent mesh complications; however, they tend to develop after a long interval. We think that the early erosion can be attributed to the operative technique; taking into regard the initially small experience in the use of the robotic system, a localized thermal injury of the vaginal mucosa during preparation may have favored this complication.

Regarding vaginal cuff insufficiency after a hysterectomy, it is known that it represents a well-studied complication in gynecologic surgery. However, high-quality data are rare, which hinders unanimous recommendations for the operative steps to be undertaken. Additionally, the available data are inconsistent about the incidence of this complication in the various forms of hysterectomy [24]. In general, the incidence of vaginal cuff insufficiency seems to be lower after a vaginal hysterectomy in comparison to laparoscopic hysterectomy [9]. The use of monopolar energy during laparoscopy and failure to correctly adapt all layers of the vagina during the suture are suggested as possible explanations.

This varying frequency of vaginal cuff insufficiency after hysterectomy in regard of the surgical route raised the question whether closing the cuff vaginally after laparoscopic hysterectomy could be beneficial. This particular consideration has been a matter of controversy in the past as a large meta-analysis in 2011 demonstrated that a vaginal route for closure is accompanied by a reduced risk of cuff insufficiency [25]. Yet, there has been concern about both the methodology and data quality of included studies, so that the Italian Society for gynecological endoscopy conducted a large multicenter prospective randomized to clarify this important consideration. This trial with 1310 patients demonstrated that the vaginal cuff insufficiency appeared more frequently after cuff closure through the vaginal route than laparoscopic closure (2.7% vs. 1%). Thus, laparoscopic closure after laparoscopic hysterectomy is recommended nowadays [26].

Regarding comparison of robotic versus laparoscopic hysterectomy, there has not been a similar high-quality data until now. However, Ucella et al. in their review with 1887 patients from 11 studies [25] demonstrated that robotic hysterectomy presents a higher risk of vaginal cuff insufficiency in comparison to the total laparoscopic hysterectomy (1.64% vs. 0.64%). Interestingly, the highest incidence of vaginal cuff insufficiency in the included studies was 5.2% and was reported in a study with a relatively low number of patients (96 patients). In our study, the incidence was with 3.8% (2/52) higher than the average reported in the review, but still remained lower than the highest reported rate in one included study [27]. This may be attributed to the low surgical experience with the daVinci system.

Other studies confirm this trend towards lower rates of vaginal cuff insufficiency with increasing number of performed procedures [24]. In a single center study with 654 robotic hysterectomies, all performed by the same surgeon, the rate of vaginal cuff insufficiency was 0.4% [28]. Dauterive and Morris reported reducing incidence of vaginal cuff insufficiency with increasing experience of the surgeon, particularly after completing the initial 25 operations [29].

There is an increased fear of complications during the initial phase of the implementation of a new technique. In our study, we could not determine such an accumulation of complications during the first procedures (Fig. 1). We think that this phenomenon has multiple contributing factors. One is the very well-tailored training program of Intuitive Surgical, which is a prerequisite for any daVinci surgeon. This program includes not only digital training modules and exercises at a pelvic trainer, but also teaching at an animal model. Additionally, trainees can familiarize with the system in an established center and are accompanied by a “proctor” during the initial operations. Thus, the initial procedures can be performed with maximum safety for the patient. Another factor was that the main surgeon during the implementation phase was highly experienced in conventional laparoscopy, which contributed to the low complication rates. An additional explanation for the evenly distributed complications during the study period was the patient selection. At the initial phase, there was a deliberate recruitment of cases with lower grade of difficulty, whereas in the later course of the program, highly complex cases were selected.

Finally, the implementation of a new operative technique poses an enormous logistical challenge for the whole team. The position of the surgeon at the console leads to absence from the operating table, thus increasing the dependence from the assistant and the rest of the team. Additionally, the missing physical proximity of the surgeon impedes the communication with the staff. Therefore, adaption of the team communication to the novel procedure and optimization with increasing experience is essential for the successful implementation of robotic surgery [30].

Our experience in Würzburg shows that adequate training of the entire team, a structured communication, and the standardization of all processes are of great importance for the smooth transition to the novel method. The structured training program with digital and pelvic trainer modules, hands on training at the animal model, and the support from experienced robotic surgeons through the initial phase of the implementation can lead to a quick adaptation to robotic surgery and allow an experienced endoscopic surgeon to perform complex procedures relatively early during the implementation of the system. Quality of the treatment and patient safety remain of utmost importance, and can be guaranteed by a structured approach during implementation.

## Conclusion

Structured proceedings make it feasible to rapidly implement robotic surgery in a German university hospital without compromising patient safety.

## Data Availability

The anonymized data that support the findings of this study are available from the corresponding author upon reasonable request.
